# Systematic review of blood diagnostic markers in colorectal cancer

**DOI:** 10.1007/s10151-018-1820-3

**Published:** 2018-07-18

**Authors:** Stella Nikolaou, Shengyang Qiu, Francesca Fiorentino, Shahnawaz Rasheed, Paris Tekkis, Christos Kontovounisios

**Affiliations:** 1 Department of Colorectal Surgery, Chelsea and Westminster Hospital, London, UK; 2 Department of Colorectal Surgery, Royal Marsden Hospital, London, UK; 3 Department of Surgery and Cancer, Imperial College, Royal Marsden Hospital, Fulham Road and Chelsea and Westminster Campus, 369 Fulham Road, London, SW10 9NH UK

**Keywords:** Biomarkers, Diagnosis, Bowel cancer, Tissue, Serum, Review

## Abstract

The purpose of this systematic review was to compare the diagnostic ability of blood markers for colorectal cancer (CRC). A systematic review of the literature for diagnostic blood markers for primary human colorectal cancer over the last 5 years was performed. The primary outcome was to assess the diagnostic ability of these markers in diagnosing colorectal cancer. The secondary outcome was to see whether the marker was compared to other markers. The tertiary outcome was to assess diagnostic ability in early versus late CRC, including stage IV disease. We identified 51 studies (29 prospective, 14 retrospective, and 8 meta-analyses). The markers were divided in broadly four groups: nucleic acids (RNA/DNA/messenger RNA/microRNAs), cytokines, antibodies, and proteins. The most promising circulating markers identified among the nucleid acids were NEAT_v2 non-coding RNA, SDC2 methylated DNA, and SEPT9 methylated DNA. The most promising cytokine to detect CRC was interleukin 8, and the most promising circulating proteins were CA11-19 glycoprotein and DC-SIGN/DC-SIGNR. Sensitivities of these markers for detecting primary colorectal carcinoma ranged from 70 to 98% and specificities from 84 to 98.7%. The best studied blood marker was SEPT9 methylated DNA, which showed great variability with sensitivities ranging from 48.2 to 95.6% and specificities from 80 to 98.9%, making its clinical applicability challenging. If combined with fecal immunochemical test (FIT), the sensitivity improved from 78 to 94% in detecting CRC. Methylated SEPT9, methylated SDC2, and -SIGN/DC-SIGNR protein had better sensitivity and specificity than CEA or CA 19-9. With the exception of SEPT9 which is currently being implemented as a screening test for CRC all other markers lacked reproducibility and standardization and were studied in relatively small population samples.

## Introduction

Colorectal cancer (CRC) is the third commonest cancer worldwide and caused 15,903 deaths in 2014 in the United Kingdom alone [1]. The stage of disease at diagnosis is the most important factor dictating survival. If the cancer is detected early, the reported 5-year survival rate is 90%, which can decrease to 14% if the disease is advanced on diagnosis [2]. The natural history of CRC is to develop from a benign adenoma, and the estimated time interval for development from normal mucosa to adenoma to invasive adenocarcinoma is 5–10 years [3, 4]. Detecting the disease early, therefore, is key to reducing mortality.

As most patients with CRC are asymptomatic or have non-specific symptoms in the early stages, it is vital to find a safe, acceptable, sensitive, specific, and cost-effective test that detects the early stage of the disease [5].

Currently, colonoscopy is the gold-standard diagnostic test to identify colonic pathology [2, 6]. A meta-analysis in 2015 by Brenner et al., showed that colonoscopy is estimated to reduce colorectal cancer incidence by 69% and mortality by 68% [7]. However, this is invasive, has low adherence, and is associated with potential risks to the patient [2]. Its alternative, virtual colonoscopy still requires bowel preparation and can cause discomfort to the patient. The risk of unnecessary radiation especially in the young is also an important disadvantage [8]. Other screening investigations include flexible sigmoidoscopy, fecal occult blood test (FOBT), and fecal immunohistochemistry test (FIT), which is a DNA-based fecal test. Although the FOBT has less than 50% sensitivity for CRC, the FIT has a reported 78% sensitivity and 96% specificity [9]. Aversion to handling stool is an important reason for the low uptake of the test. Only 58% of patients who are sent the FOBT return a sample. In the UK, the introduction of the DNA-based fecal test, which is easier to use, is expected to increase uptake to 75% by 2020, with the challenge of obtaining a sample for testing from stool still remaining [10].

Blood-based markers in current use, such as carcinoembryonic antigen (CEA) and cancer antigen (CA) 19-9, are for surveillance and for monitoring response to treatment but have a low sensitivity and specificity ranging from 40 to 70% and 73 to 90%, respectively, making them unsuitable as screening or diagnostic markers [5, 10] A more recently proposed marker, which is commercially available, is methylated septin 9. This is a molecular-based blood test whose reported accuracy in the literature has been variable. A recent meta-analysis by Yan et al. has shown sensitivity of 76% and specificity 87% making it comparable to the DNA-based fecal test [11].

The purpose of this study is a systematic review of the literature on diagnostic biomarkers in blood or tissue over the last 5 years in colorectal cancer.

## Materials and methods

### Inclusion criteria

- Primary human studies and meta-analyses in the last 5 years, which assessed serum or tissue markers’ diagnostic ability in CRC.

### Exclusion criteria

- Studies looking at familial or inherited CRC
- Studies with less than 100 patients overall
- Animal or in vitro studies
- Studies that did not specify sensitivity or specificity of the markers.

#### Search strategy

**Fig. 1:**
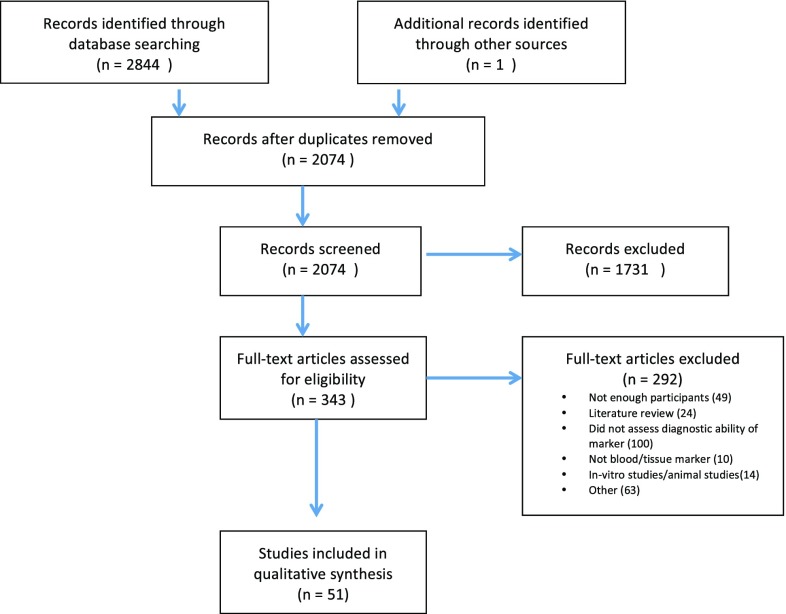
PRISMA diagram of systematic review

An electronic search of PubMed, EMBASE, Cochrane, and ISI Web of Science was performed for the relevant studies between January 2013 and December of 2017 using the following terms: (marker OR biomarker) AND (serum OR blood OR tissue) AND (diagnosis OR screening) AND (colorectal OR colon OR bowel or rectal) AND (cancer OR carcinoma OR neoplasia). There were no language restrictions and duplicates were removed. After reviewing the title and abstract of the studies, as per the Preferred Reporting Items for Systematic Reviews and Meta-Analyses (PRISMA) flowchart, the relevant manuscripts were selected for full-text review (Fig. 1). One additional relevant article was added. A 5-year timeframe was chosen to provide a summary of the recent advances in biomarkers and of the ones that have potential for future use.

#### Data extraction

All original studies and meta-analyses which assessed diagnostic ability of markers were included. Data extracted from each full-text manuscript were as follows: authors, publication year, whether it was serum or plasma or whole blood or tissue, area under the curve, sensitivity, and specificity of detecting primary CRC and if compared to another standard test and cutoff values. If the AUC or sensitivity or specificity was not specifically determined the study was excluded from the analysis.

#### Study outcomes

The primary outcome was to compare the diagnostic ability of the markers studied in blood or tissue. The secondary outcome was to see whether the marker was compared to other markers. The tertiary outcome was to assess the diagnostic ability of the marker in early versus late CRC.

## Results

The literature search over the last 5 years yielded 2844 papers from which 51 studies were eligible for inclusion in the review (Fig. 1).

The markers were divided in broadly four groups: nucleic acids (RNA/DNA/messenger RNA/microRNAs), cytokines, antibodies, and proteins. The nucleic acid category was further subdivided into single microRNA, panel of microRNAs, and a separate group with RNA, DNA, and messenger RNA. They are summarized in Tables 1, 2, 3, 4, 5 and 6. Overall, 29 prospective studies, 14 retrospective studies, and 8 meta-analyses were included.

**Table 1:** Single miRNA markers

Author (year)	Type of study	Marker	No. of patients	Blood	CRC versus control			Cutoff value	Compared to another marker	Early CRC versus control	Tissue
					AUC	Sensitivity	Specificity				
Li et al. (2016) [34]	Prospective	− 29b	200 CRC 400 control	Plasma	0.743	61.4%	72.5%		No	No	Yes (sens 81.6%, spec 84.9%)
Basati et al. (2016) [35]	Retrospective	− 29b	55 CRC 55 controls	Serum	0.87	77%	75%	0.66	No	No	No
		− 194			0.85	72%	80%	1.08			
Imaok a et al. (2016) [11]	Retrospective	− 1290	211 CRC 56 adenomas, 57 controls	Serum	0.830	70.1%	91.2%	ND	No	Yes (level higher in IV compared to I–III + compared adenoma versus control)	Yes
Zhi et al. (2015) [36]	Meta-analysis (5 studies)	− 29a	281 CRC 299 healthy		0.9128	Pooled sens 59%	Pooled spec 89%	1.33 0.545 7.2, 2 studies not specified	No	ND	No
Xu et al. (2015) [37]	Meta-analysis (7 studies)	− 21	676 CRC 417 controls	ND	0.86	Pooled sens 75%	Pooled spec 84%	3.59 0.0043 0.0019 3.703 1.08 1.49, 1 study not specified	2 studies: miR-16 U6RNA, cel-MIR-39, miR-16, miR-451, RNU6B	ND	No
Lv et al. (2015) [38]	Retrospective	− 155	146 CRC 60 controls	Serum	0.776	58.2%	95%	1.102	No	High levels were correlated to diff/TNM but no sens/spec	No
Chen et al. (2015) [39]	Prospective	− 106a	100 CRC 79 cancer-free	Blood	0.605	32%	83.54%	3.52	No	ND	No
		− 20a			0.590	46%	73.42%	2.44			
Zhang et al. (2014) [40]	Meta-analysis (6 studies)	− 21	1071 patients	3-Serum, 3-Plasma	0.76	81%	81%	ND	No	ND	Yes
Yang et al. (2014) [41]	Meta-analysis (6 studies)	− 92a	521 CRC 379 healthy	ND	0.772	76%	64%	0.000,1 7 02972 1.231 2.87 240	No	ND	No
Xu et al. (2014) [42]	Prospective	− 375	94 CRC 46 healthy	Plasma	0.7489	76.92%	64.63%	0.4852	No	No	Yes
Nonaka et al. (2014) [43]	Prospective	− 199a-3p	84 CRC 32 non-cancer	Serum	0.644	47.6%	75%	0.0010	Yes (miR-21 AUC 0.675, sens 54.7%, spec 84.4%)	No	Yes

*AUC* area under the curve, *CRC* colorectal cancer, *ND* not discussed, *TNM* TNM classification (*T* size of tumor, *N* lumph nodes involved, *M* metastasis), *sens* sensitivity, *spec* specificity

**Table 2:** miRNA panel markers

Author (year)	Type of study	Panel of markers	No. of patients	Blood	CRC versus control			Compared to CEA/CA19-9 (sens/spec)	Early CRC versus control	Tissue
					AUC	Sensitivity	Specificity			
Zhu et al. (2017) [44]	Prospective	-19a-3p, miR-21-5p, -425-5p	196 CRC 138 controls	Serum	0.830	ND	ND	No	No	Yes
Vychytilova-Faltejskova et al. (2016) [45]	Prospective	-23a-3p -27a-3p -142-5p -376c-3p	103 CRC 100 controls	Serum	0.922	89	81	Yes (CEA 47% sens, CA19-9 sens 27%)	Yes (I/II: AUC 0.877 sens 81 spec 81)	Yes
Fang et al. (2015) [46]	Retrospective	-24, -320a, -423-5p	111 CRC 59 adenomas, 24 polyps 29 IBD 130 healthy	Plasma	0.899	92.79%	70.77%	Yes (CEA sens 20.37%, spec 95%, CA19-9 sens 20.37%, spec 93.08%)	I/II: AUC 0.99 sens 90.79%, spec 70.77%	No
Wang et al. (2014) [47]	Retrospective	-21, Let-7 g, -31, -92a, -181b, -203	83 CRC, 59 controls	Serum	0.923	ND	ND	Y (CA19-9 AUC 0.598, CEA, AUC 0.649)	No	No
Zhang et al. (2013) [48]	Retrospective	-200c, -18a	78 CRC, 86 normal	Plasma	0.839	84.6%	75.6%	Y (-18a: sens 73.1%, spec 79.1%, -200c: sens 64.1%, spec 73.3%)	No	Yes
Luo et al. (2013) [49]	Prospective	-18a, -20a, -21-29a, -92a, -106b, -133a, -143, -145, -181b, -342-3p, 532-3p	80 CRC 144 neoplasm-free	Plasma	0.745	ND	ND	ND	Yes (no major diff. between stages was found)	No

If diagnostic values in any paper were better in a panel rather than the individual markers, the panel was tabulated and not the individual ones

*AUC* area under the curve, *CRC* colorectal cancer, *CA19-9* Cancer antigen 19-9, *CEA* carcinoembryonic antigen, *diff*. difference, *IBD* inflammatory bowel disease, *ND* not discussed, *sens* sensitivity, *spec* specificity

**Table 3:** RNA/DNA/messenger RNA markers

Author (year)	Type of study	Marker	Number of patients	Blood	CRC versus control					Cutoff value	Compared to CEA/CA19-9	Early CRC versus control	Tissue
					AUC		Sensitivity (%)		Specificity (%)				
Nagai et al. (2017) [50]	Retrospective	LINE-1 hypomethylation index in cfDNA	114 CRC 53 control	Plasma	0.81		65.8	90		0.360	cfDNA concentration (less accurate than cfDNA LHI)	Sens 63.2% spec 90% for early CRC	No
Nian et al. (2017) [14]	Meta-analysis (25 studies)	Methylated SEPT 9	2975 CRC, 6952 adenoma	ND	0.88		71	92		Range of values (9 studies not specified)	N (* combined to FIT: 94% sens, 68% spec)	Sens 45% stage I, 70% stage II	No
Wu et al. (2015) [15]	Prospective	Long non-coding RNA NEAT1_v1	100 CRC 100 controls	Whole blood	0.787		69	79		ND	No	Yes (no difference between mean values in early stage (I/II) compared to late stage III/IV)	Yes (no correl ation betwe en CRC tissue and whole blood)
		NEAT1_V2			0.871		70	96			No		
Pedersen et al. (2015) [51]	Prospective	methylatedBCAT1 + IKZF1	129 CRC, 685 adenoma, 1291 no neoplasia	Plasma	ND	66		94		ND	No	Yes (I/II sens 56%, III/IV 79%)	No
Jin et al. (2015) [26]	Retrospective	Septin 9	135 CRC 169 polyps, 91 healthy	Plasma	ND	74.8		87.4		ND	No (but compared to FIT: sens 58%, spec 82.4%)	No	No
Hao et al. (2014) [16]	Prospective	ALU115 of circulating free DNA	104 CRC 63 polyps, 110 normal	Serum	0.85	69.23%		99.09%		694 ng/ml	CEA (AUC 0.78, sens 42.31%, spec 100%). CEA + ALU115 + ALU247/1 15 = SENS 85.57% SPEC 97.27%	Yes (levels significantly different between primary CRC and polyps/controls)	No
		ALU247/115 of serum DNA			0.89	73.08		97.27		0.52 ng/ml			
Qi et al. (2013) [52]	Prospective	Alu-based cell-free DNA	31 CRC 30 polyp 92 healthy	Serum	0.904	64.5		98.9		634.9 ng/mL	Yes (CEA AUC 0.681, CA19-9 AUC 0.651-polyp versus CRC)	No	No
Oh et al. (2013) [17]	Prospective	SDC2 methylation in DNA	131 CRC 125 healthy	Serum	ND	87		95.2		0.936	No	Sens 92% stage I	Yes
Rodia et al. (2016) [53]	Meta-analysis	TSPAN 8	67 CRC, 67 control	Whole blood	0.751	83.6		58.2		N/D	No	No	No
		LGALS4			0.746	82.1		61.2		N/D			
		COL1A2			0.718	73.1		59.7		N/D			
		CEACAM 6			0.632	65.7		61.2		N/D			
		TSPAN8 + LGALS 4			0.862	92.54		67.16		N/D			
Li et al. (2016) [34]	Retrospective	mRNAM	200 CRC, 200 benign disease	Tissue	0.823	89.6		84.5		0.3562	No	No	Yes
					0.802	80.5		76.5		0.3023			
					0.814	86.5		82.9		0.3243			
Khales et al. (2015) [19]	Prospective	mRNA	51 CRC, 60 healthy	Serum	0.981	96.1		95		14,000 copies/1 ml of blood	Yes (CEA-correlated to SALL4)	Yes (no diff between early CRC)	Yes
Wang et al. (2014) [47]	Prospective	mRNA	92 CRC, 60 healthy	Serum	0.855	84.8		80		0.128	CEA (AUC 0.691)	No I, 28.8% Stage II	No
Qi et al. (2013) [52]	Prospective	Alu-based cell-free DNA	31 CRC 30 polyp 92 healthy	Serum	0.904	64.5		98.9		634.9 ng/mL	Yes (CEA AUC 0.681, CA19-9 AUC 0.651-polyp versus CRC)	No	No
Oh et al. (2013) [17]	Prospective	SDC2 methylation in DNA	131 CRC 125 healthy	Serum	ND	87		95.2		0.936	No	Sens 92% stage I	Yes
Rodia et al. (2016) [53]	Meta-analysis	TSPAN 8	67 CRC, 67 control	Whole blood	0.751	83.6		58.2		N/D	No	No	No
		LGALS4			0.746	82.1		61.2		N/D			
		COL1A2			0.718	73.1		59.7		N/D			
		CEACAM 6			0.632	65.7		61.2		N/D			
		TSPAN8 + LGALS 4			0.862	92.54		67.16		N/D			
Li et al. (2016) [34]	Retrospective	mRNAM	200 CRC, 200 benign disease	Tissue	0.823	89.6		84.5		0.3562	No	No	Yes
					0.802	80.5		76.5		0.3023			
					0.814	86.5		82.9		0.3243			
Khales et al. (2015) [19]	Prospective	mRNA	51 CRC, 60 healthy	Serum	0.981	96.1		95		14,000 copies/1 ml of blood	Yes (CEA-correlated to SALL4)	Yes (no diff between early CRC)	Yes
Wang et al. (2014) [47]	Prospective	mRNA	92 CRC, 60 healthy	Serum	0.855	84.8		80		0.128	CEA (AUC 0.691)	No	No

*ALU115/247* Arthrobacter Luteus 115/247, *AUC* area under the curve, *BCAT1* branched chain amino acid transaminase 1, *CA19-9* cancer antigen 19-9, *CEA* carcinoembryonic antigen, *CEACAM6* carcinoembryonic antigen-related cell adhesion molecule 6, *cfDNA* circulating free DNA, *COL1A2* collagen alpha-2 (I), *CRC* colorectal cancer, *FIT* fecal Immunochemical test, *IKZF1* Ikaros family zinc-finger protein 1, *LGALS4* Galectin-4, *LINE1* long-interspersed nuclear element, *LH1* LINE-1 hypomethylation index, *mRNA* messenger RNA, *NEAT1V_1/2* Nuclear-enriched abundant transcript1_variant1/2, *ND* not discussed; *ng/mL* nanogram per milliliter, *SALL4* Sal-like protein4, *sens* sensitivity, *SEPT9* Septin 9, *spec* specificity, *SDC2* Syndecan-2, *TSPAN8* Tetraspanin 8

**Table 4:** Cytokine markers

Author (year)	Type of study	Marker	Number of patient s	Blood	CRC versus control			Cutoff value	Compared to CEA/CA19**-**9	Early CRC versus control	Tissue
					AUC	Sensitivity (%)	Specificity (%)				
Wang et al. (2017) [54]	Retrospective	Macrophag e Inhibitory Cytokine (MIC-1/GDF15)	473 CRC, 25 polyps, 489 controls	Serum	0.866	43.8	96.7	1000 pg/mL	CEA (AUC 0.728, sens 36.6%) CEA + MIC-1 = AUC 0.886, sens 72.7% 89% specificity	AUC 0.843 sens 38.5%	No
Xu et al. (2016) [55]	Meta-analysis (7 for diagnostic)	IL-6	687 CRC, 392 controls	ND	0.79	72	74	2.14, 3.06 4.24, 6.70 and 3 studies ND	n/a	n/a	No
Zheng et al. (2015) [56]	Prospective	Growth-related gene product beta 1	123 CRC, 125 non-tumor, 88 healthy	Serum	0.834	56.1	95.31	105 pg/mL	CEA AUC 0.739 CA19-9 AUC 0.676	If combine d to CEA, then detects early CRC 22.2% from 5.6% for stage I, 66.7% from 41% for stage II	No
Xia et al. (2015) [20]	Meta-analysis (5 diagnostic)	IL-8	725 total	ND	0.92	70	91	17.71 pg/mL, 39.5 pg/mL, 44.26 pg/mL, 8.83 pg/mL	ND	No	No

*AUC* area under the curve, *CA19-9* cancer antigen 19-9, *CEA* carcinoembryonic antigen, *CRC* colorectal cancer, *IL* interleukin, *N/a* not applicable, *ND* not discussed; *pg/mL* picogram per milliliter, *sens* sensitivity, *spec* specificity

**Table 5:** Antibody markers

Author (year)	Type of study	Marker	Number of patients	Blood	CRC versus control			Cutoff value	Compared to CEA/CA19-9	Early CRC versus control	Tissue
					AUC	Sensitivity (%)	Specificity				
Kunizaki et al. (2016) [57]	Prospective	A Anti-p53	170	serum	ND	30.6	ND	ND	CEA and anti-p53: detection	31.9%	No
Chen et al. (2016) [58]	Prospective	Anti-p53	49 CRC 99 AA, 29 non-advanced adenomas, 224 controls	Serum	ND	18	90%	ND	No	26% sensitivity for early CRC	No
		Anti-IMPDH2									
		Anti-MDM2									
		Anti-MAGEA4									
Wang et al. (2016) [59]	Prospective	FnIgA	258 CRC 150 benign, 200 healthy	Serum	0.704	36.43	92.7%	0.45	Yes CEA and CA19-9. If CEA + CA19-9 + anti FnIgA: AUC 0.858, sen 54.65% spec 96.6%	Sens 27.7% spec 96.21%	No
		FnIgG			0.645	77.52	46.94%	0.42		ND	

*ND* not discussed, *AUC* area under the curve, *CRC* colorectal cancer, *AA* advanced adenoma, *AUC* area under the curve, *CA19-9* cancer antigen 19-9, *CEA* Carcinoembryonic antigen, *CRC* colorectal cancer, *FnIgA/G* fusobacterium nucleatum immunoglobulin A/G, *Ig* immunoglobulin, *IMPDH2* inosine monophosphate dehydrogenase 2, *MAGEA4* melanoma-associated antigen A4, *MDM2* Mouse double minute 2 homolog, *ND* not discussed, *sens* sensitivity, *spec* specificity

**Table 6:** Protein markers

Author	Type of study	Marker	Number of patients	Blood	CRC versus control			Cutoff value	CEA/CA19**-**9	Early CRC versus control	Tissue
					AUC	Sensitivity	Specificity				
Fei et al. (2017) [60]	Retrospective	RBP4	402 CRC, 218 Normal	Serum	0.852	74.9%	81.7%	26.7 µg/mL	Y (CEA: AUC 0.817, CA19-9: AUC 0.634)	ND	No
		THBS2			0.794	64.9%	87.1%	14.85 ng/mL			
		RBP4 + CEA			0.927	80.8%	91.2%	ND			
Li et al. (2016) [21]	Prospective	TFF 3	127 CRC, 77 controls (35 polyps, 42 controls)	Serum	0.889	74.2%	94.8%	5.591 ng/m L	Yes (CEA: AUC 0.715 sens 62.2%, spec 72.7%)	Yes (TFF3 significantly higher in stage I CRC comp to controls but not diff. if polyps)	No
Werner et al. (2016) [9]	Retrospective	CEA Ferritin Seprase Osteoponti n Anti-p53	36 CRC 420 advanc ed adenom a, 1200 controls	Serum	0.78	44%	90%	ND	Yes (CEA sens 50%, spec 90%)	Yes (early cancers were detected at least as well as late-stage)	No
		CEA + anti-p53			0.85	58%	90%				
Wang et al. (2016) [61]	Prospective	COL3A1	86 CRC 21 enteritis, 3 polyps, 68 normal	Plasma Epithelial tissue	0.92 0.975	98.8% 95.2%	69.1% 91.1%	54.23 ng/m L	Yes (CEA: AUC 0.791, sens 70.2%, spec 73%)	Yes	Yes (mRNA)
Rho et al. (2016) [22]	Retrospective	BAG4 IL6ST** VWF EGFR BAG4 IL6ST VWF CD44	60 CRC 60 adenom as, 30 control	Plasma/Serum	0.81 0.79	40.9% 42.44%	90%	ND	No	No	Yes
Overholt et al. (2016) [22]	Prospective	CA11-19	131 CRC, 65 polyps, 182 benign disease, 103 controls	Serum	ND	98%	84%	6.4 units/mL	No	No	No
Gezer et al. (2015) [62]	Prospective	Trimethylations of lysine 9 on histone 3 (H3K9me3) Trimethylations of lysine 20 on histone 4 (H4K20me3)	63 CRC 40 cancer-free	Plasma	No significant difference between CRC and controls 0.715	ND 14.3%	Yes 95%	No	No		
		H3K27me3			0.620	17.5%	95%				
		H3K27me3 + H4K20me3			0.769	28.6%	95%				
Xue et al. (2015) [63]	Prospective	Zinc-alpha-2-glycoprote in (AZGP1)	120 CRC, 40 healthy	Serum	0.742	55.8%	85%	2297.71 ng/ mL	Yes	No	Yes
		AZGP1 + CEA + CA1 9-9			0.805	67.5%	82.5%				
Wang et al. (2015) [64]	Prospective	Angiopoeti n-2	98CRC 90 healthy	Serum	0.859	79.3%	82.4%	2710 pg/mL	No	No	No
Storm et al. (2015) [65]	Retrospective	CL-L1 M-ficolin MAp44	99CRC 196 adenom as, 696 no cancer	Serum	0.68	36%	83%	ND	No	No	No
Fung et al. (2015) [66]	Prospective	IGFBP2 DKK3 PKM2	98 CRC 99 controls	Serum	0.91	73%	95%	ND	No (but compared to FOBT and FIT and equivalent)	Yes (stage I: sens 59%, II 84%, III 71%, IV 78% for spec 95%)	No
Sole et al. (2014) [67]	Retrospective	COL10A1	80CRC 23 adenom a, 77 controls	Serum	0.76	63%	85%	208 ng/mL	ND	No	Yes
Shin et al. (2014) [68]	Retrospective	Melanotra nsferrin	228 CRC, 20 polyps, 77 healthy	Plasma	0.723	48.2%	92.5%	ND	Yes (CEA, PAI-1) AUC of regressed TRFM-PAI1-CEA 0.821, sens 67.5%, spec 90%	Yes	Yes
Shirahata et al. (2014) [69]	Prospective	Vimentin methylation	242 CRC 25 healthy	Serum	ND	32.6%	ND	0.0485 ng/mL	Vimentin + CEA + CA19-9 = sens 55.6%	Sens 57.1% for stage 0, 30.6%	No
Jiang et al. (2014) [24]	Prospective	sDC-SIGN & sDC-SIGNR	182 CRC, 101 healthy	Serum	0.9885	98.7%	94.8%	sDC-SIGN 2.226 μg/mL/sDC-SIGNR 222.7 ng/m L	Yes (CEA sens 29.22%, CA19-9 14.67%)	Yes(better at detecting early CRC compared to CEA/CA19-9; DC-SIGN sens 81.33, spec 55.56%; DC-SIGNR sens 48.65%, spec 92.5%	Yes
Wang et al. (2013) [70]	Prospective	Kininogen	140 CRC, 80 adenom as, 85 healthy	Serum	0.706	63.64%	65.88%	162.99 μg/ml	Yes (CEA: AUC: 0.695 sens 38.46%, spec 85.88%)kininogen-1 or CEA: sens 79.92%, spec 58.82%	Yes	Yes

*AUC* area under the curve, *AZGP1* zinc-alpha-2-glycoprotein, *BAG4* BCL2-associated athanogene 4, *CA11-19* cancer antigen 11–19, *CA19-9* cancer antigen 19-9, *CD44* cluster of differentiation 44, *CEA* carcinoembryonic antigen, *CL-L1* Collectin-liver 1, *COL3A1* collagen type III alpha 1, *COL10A1* Collagen type X alpha 1, *CRC* colorectal cancer, *DKK3* Dickkopf 3, *EGFR* epidermal growth factor receptor, *IGFBP2* insulin-like growth factor binding protein 2, *ILGST* interleukin-5 receptor subunit beta(**this is a cytokine receptor), *MAp44* Mannan-binding lectin-associated protein 44, *mRNA* messenger RNA, *ND* not discussed, *ng/mL* nanogram per milliliter, *PAI-1* plasminogen activator inhibitor 1, *PKM2* pyruvate kinase M2, *RBP4* retinol binding protein 4, *sDC-SIGN* serum dendritic cell-specific ICAM-3 grabbing nonintegrin, *DC-SIGNR* DC-SIGN-related protein, *sens*. sensitivity, *spec* specificity, *TFF3* Trefoil factor 3, *TRFM* Melanotransferrin, *THBS2* Trombospondin 2, *µg/mL* micrograms per milliliter, *VWF* Von Willebrand factor

### Nucleic acids (Tables 1, 2, 3)

#### MicroRNAs (Tables 1, 2)

These are small non-coding RNA particles, which regulate gene expression by binding to messenger RNA (mRNA) and affecting protein translation or gene expression. They are thought to act as either tumor suppressor genes or oncogenes. In the literature, they have been investigated singly or in panels to assess their diagnostic and prognostic capabilities. A study done by Imaoka et al. in 2016, reported that mi-1290 showed promise as a diagnostic marker. Mi-1290, is thought to promote epithelial mesenchymal transition (EMT), proliferation and has metastatic potential. The study investigated 324 patients and showed a sensitivity of 70.1% and specificity of 91.2% in detecting CRC. Its sensitivity in detecting adenomas compared to controls was 46.4% and specificity 91.2%, therefore inadequate to be used as a screening test on its own [11].

Table 1 shows studies that investigated a single miRNA in colorectal cancer and Table 2 shows the studies that investigated panels of these markers.

A meta-analysis performed by Zhang et al. for mi-21 showed a sensitivity and specificity of 81% [12] whilst Xu F et al.’s meta-analysis for the same marker in 2015, showed a pooled sensitivity of 75% with specificity of 84%. Among the original studies, the most promising was for mi-1290, which showed an AUC of 0.830, sensitivity of 70.1% and specificity of 91.2% [11].

Assessing different combinations of miRNAs had variable results. Fang et al. investigated a panel of three markers (mi-24, mi-320a, and mi-423-5p) which showed an overall sensitivity of 92.79% and specificity of 70.77% in detecting CRC and also showed high sensitivity and specificity in detecting early cancer [13].

#### RNA/DNA/messenger RNA (Table 3)

Septin 9, which has been well studied and is now commercially available [Epi proColon 2.0 (Epigenomics), mS9 (Abbott Molecular), ColoVantage (Quest Diagnostics)], has had disputed results. A meta-analysis by Nian et al. published in 2017, included 25 studies, of which only 2 showed a low risk of bias. Twenty-one studies excluded “difficult-to-diagnose” patients and seven studies did not specify thresholds used. The pooled sensitivity was 72% and specificity of 92% which if combined with FIT can increase up to 94% sensitivity, with a decreased specificity of 68% [14]. It also highlighted that the sensitivity for stage I disease was 45% and for polyps was 15% which makes it rather poor for a screening test.

A prospective analysis by Wu et al. investigated long non-coding RNA nuclear-enriched abundant transcript variants 1 and 2 (NEAT_v1 and NEAT_v2). Non-protein coding RNAs are greater than 200 nucleotides and constitute more than 70% of the genome. Non-protein coding RNA nuclear-enriched abundant gene 1 has 2 transcripts: NEAT1_v1 and NEAT1_v2. NEAT1_v2 showed a 70% overall sensitivity and 96% specificity in detecting CRC from controls, although the mean value in early versus late CRC was not significantly different [15]. Further study with larger cohorts and in different types of cancer is required to further validate this marker.

Hao et al., investigated ALU sequences in circulating free DNA, which are the most active sequences in the human genome [16]. ALU115, ALU247/115, and CEA, had a sensitivity of 85.57% and specificity of 97.27% in detecting CRC [16].

SDC2 is an integral membrane protein and is known to participate in cell migration and proliferation of cells. The SDC2 gene is expressed in mesenchymal but not epithelial colonic cells. It is also expressed in pancreatic epithelial cells. SDC2 methylation of DNA in a prospective study by Oh T et al. in 2013 showed sensitivity of detecting early CRC of 92% although this needs further validation in a larger study [17].

Messenger RNA conveys information from DNA to protein products of the genes expressed. The studies looking into mRNA have many flaws in the study design and description of control groups and many do not investigate the sensitivity and specificity of the marker in detecting early CRC. Cyclin E, p27kipl, and ki-67, investigated by Li et al. in a retrospective study, showed sensitivities and specificities around 80% [18]. However, this was only measured on tissue and not correlated to blood markers.

SALL4, a zinc-finger transcription factor, was evaluated in a prospective study by Khales et al. in 2015 and showed sensitivities of 96.1% and specificity of 95% [19]. This transcription factor is also found in other cancers and needs further validation in a larger cohort of patients which include polyps [19].

#### Cytokines (Table 4)

These are small secreted proteins, which can have autocrine or paracrine effects. Types of cytokines include chemokines (e.g., interleukin-8), lymphokines (e.g., interleukin-6), and interferons. They are released by a variety of cells including macrophages, T cells, B cells, and mast cells, and have been implicated in inflammatory and neoplastic diseases. The most promising was interleukin-8, which showed a sensitivity of 70% and specificity of 91% in detecting CRC in a meta-analysis conducted by Xia WJ et al. in 2015 [20]. Interleukin-8 is a chemokine thought to be involved in cancer progression and promotes angiogenesis, proliferation and migration of the cancer cells [20]. The study included 5 diagnostic studies with 725 participants. They were all high quality studies and if 1 study, by Burger et al., was excluded, the heterogeneity was significantly reduced. Limitations to this study were that it includes a relatively small selection of studies, the cutoffs of the different studies varied and a subgroup analysis could not be performed [20].

#### Antibodies (Table 5)

There were only three studies in the last 5 years that investigated antibodies, which fulfilled the inclusion criteria. None of the studies showed promising enough results for their use in diagnosis of CRC.

#### Proteins (Table 6)

In this category, the more promising proteins were trefoil factor (TFF)3 [21], CA11-19 [22], a combination of insulin-like growth factor binding protein 2 (IGFBP2), Dickkopf-3(DKK3), and pyruvate kinase M2 (PKM2) [23] and DC-SIGN/DC-SIGNR [24].

TFF3 belongs to a TFF family, which consists of three stable secretory proteins: TFF1-3. TFF3 is secreted by goblet cells of the intestine and to a lesser extent in the salivary glands, breast, and respiratory tissue. It is thought to promote invasion of cells by acting directly on the cells and indirectly on the vasculature. Results from the study by Li et al. showed a sensitivity of 74.2% and specificity of 94.8%; however, the level of this marker in polyps is not significantly different from that in the CRC cohort, making this less likely to be a useful diagnostic test [21].

CA11-19 is a 701 amino acid glycoprotein which showed very promising results in detecting CRC in a study by Overholt et al [22]. It showed a sensitivity of only 40% in detecting adenomatous polyps, but again a larger study is needed to include more CRC and more patients with polyps. Other limitations for this study were that only one center was included and the authors indicated a larger multi-center study was being planned [22].

Fung KY et al. investigated a combination of IGFBP2DKK3 and PKM2 [23] which have been implicated in proliferation, migration, and angiogenesis of cancer cells. The study showed a sensitivity of 73% and specificity of 95% in detecting CRC [23]. The sensitivity of detecting early stage cancer was moderate with 59% in stage I and 84% in stage II. This again needs further validation in a larger study, which includes patients with polyps.

DC-SIGN and DC-SIGNR are membrane-bound C type lectins. DC-SIGN is found on the surface of dendritic cells in the colon but also in the placenta, cervical mucosa and uterus. DC-SIGNR is found on the endothelial cells in the placenta, liver, and lymph nodes. Jian YM et al. investigated the serum level of DC-SIGN and DC-SIGNR, showing very high sensitivity and specificity in detecting CRC from healthy controls in a 290-patient cohort. The markers (DC-SIGN and DC-SIGNR) were separately analyzed for their sensitivity of detecting early CRC (stage I–III) and this was higher compared to CEA/CA19-9, yet polyps were not investigated [24].

### Selection of markers for outcome study

From our review the following markers were found to have a sensitivity ≥ 70% and specificity ≥ 90%: interleukin 8 [25], NEAT_v2, SDC2 methylation of DNA [17], SEPT9 [14, 26], CA11-19 [22] and DC-SIGN/DC-SIGNR [24]. This cutoff sensitivity and specificity were used because they will discriminate markers which show promise in detecting CRC, and are comparable to the currently available biomarkers. The primary, secondary, and tertiary outcomes for these markers are as follows.

#### Diagnostic ability summary

Interleukin 8 (sensitivity 70%, specificity 91%, AUC 0.92), NEAT_v2 (sensitivity 70%, specificity 96%, AUC 0.871), SDC2 methylation of DNA (sensitivity 87%, specificity 95.2%), SEPT9 (sensitivity 71%, specificity 92%), CA11-19 (sensitivity 98%, specificity 84%), and DC-SIGN/DC-SIGNR (sensitivity 94.8%, specificity 98.7%, AUC 0.9885).

#### Comparison studies summary

The DC-SIGN/DC-SIGNR was compared to CEA (sensitivity 29.22%) and CA 19-9 (sensitivity 14.67%) and had better sensitivity (98.7%) and specificity (94.8%). SEPT9 in combination with fecal immunochemical test (FIT) improved sensitivity to 94% from 71% but decreased specificity to 68% from 92% in a recent meta-analysis by Nian et al [14]. Compared to FIT alone, Jin and colleagues showed that septin 9 showed a sensitivity and specificity of detecting CRC of 74.8 and 87.4%, respectively, whereas FIT alone had 58% sensitivity and 82.4% specificity [26]. CA 11-19, SDC2 methylation of DNA, NEAT1_v1 and Il-8 were not compared to any other marker.

#### Early stage detection summary

The tertiary outcome was assessed in SDC-SIGN/SDC-SIGNR, SEPT9, and SDC2 methylation of DNA and NEAT1_v1. NEAT1_v1 showed no difference in the mean value of the marker in early compared to late cancers. For SEPT9, sensitivity of detecting stage I CRC was 45% and 70% for stage II [14]. SDC2 methylation of DNA showed a sensitivity of 92% for detecting stage I CRC [17]. Finally, SDC-SIGN showed sensitivity and specificity of detecting early CRC of 81.33% and 55.56%, respectively [24].

## Discussion

Investigation of biomarkers for the diagnosis of CRC can have a significant effect on its prognosis. Ransohoff described the search for a non-invasive biomarker as the “Holy Grail of cancer biomarker research” [27].

The currently used screening tests are either too uncomfortable, costly and potentially hazardous or have a low compliance rate due to patients’ aversion to sampling stool.

This review has highlighted the large numbers of markers being investigated and yet there is a lack of well-designed studies to investigate their use in diagnosis of CRC. There is also a lack of follow-up studies for many markers which have shown promise.

One of the first blood tests brought to clinical use for screening is called ColonSentry™. This measures mRNA in a 7-gene panel (ANXA3, CLEC4D, LMNB1, PRRG4, TNFAIP6, VNN1, and IL2RB) [27]. The sensitivity and specificity for ColonSentry for detecting CRC is 72 and 70%, respectively [27]. Retrospective studies on methylated SEPT9, have reported sensitivities, which range from 52 to 72% and specificities which range from 90 to 95% in detecting CRC [28, 29]. However, methylated SEPT9 has also been studied in a screening population as part of the PRESEPT trial in the USA and Germany, showing overall sensitivity of 48.2% and specificity of 91.5% for detecting CRC [30]. The difference between the PRESEPT and the other studies was that the first investigated the screening capacity of asymptomatic patients rather than symptomatic ones.

The markers we identified as promising included the following: interleukin 8 [25], NEAT_v2, SDC2 methylation of DNA [17], SEPT9 [14, 26], CA11-19 [22] and DC-SIGN/DC-SIGNR [24]. However, none were ideal and many had limitations that need addressing. Meta-analysis had been performed for only two markers (IL-8 and SEPT9) and the first included a small number of studies with variable cutoff values and a subgroup analysis could not be performed. As for the meta-analysis for SEPT9 published by Nian et al. in 2017, only two studies showed a low risk of bias. A total of 25 studies were included and most of them used the 2/3 positive result (known as the 2/3 algorithm) of the Epipro Colon assay. The pooled sensitivity was 71% and specificity 92% for generation 1 Epipro Colon assay and 76% and 94%, respectively, for generation 2 assay. The diagnostic value was highest for stage IV disease with a sensitivity of 79% specificity of 93%. However, 12 of the studies showed that SEPT9 has a sensitivity of 15 and 5% in detecting adenomas and polyps, respectively, and pooled sensitivity for larger size (> 1 cm) polyps or adenomas was 23%, making it a less than ideal screening marker. The studies which combined FIT with SEPT9 showed a higher sensitivity (94%) but lower specificity (68%). A recent retrospective study by Fu and colleagues also found that the 1/3 positive results (known as the 1/3 algorithm) of Epipro Colon Assay 2.0, is more sensitive in detecting early CRC (sensitivity of 69.6% for stage I) although still poor at detecting polyps and adenomas (sensitivity 16.8% from 7.9% using the 2/3 algorithm) and therefore, may be useful as an early cancer screening test [31]. Although SEPT9 shows promise, there is a large heterogeneity in the study results, which may be attributed to many factors including gender, race, age, assay method, and other environmental factors so larger prospective studies are required to further verify its diagnostic potential. SDC-SIGN, SDC-SIGNR, SEPT 9, and SDC2 methylation of DNA also showed a better detection rate in early versus late CRC compared to CEA. However, the sensitivities and specificities were still too low to have any true value in diagnosing CRC.

The rest of the studies did not clearly define their control groups, many including polyps in the control group and others not including them at all. Patient cohorts were mixed between symptomatic and asymptomatic individuals, which affected the results. Furthermore, not all studies assessed their markers’ ability to detect early CRC, an important factor in a diagnostic/screening marker. Variability of cutoff values, method of analysis of the markers, and timing of sample taking can also have a significant impact on the heterogeneity of the results. Inclusion of positive controls is also important as many of these markers, test positive in other cancers or diseases.

The cost-effectiveness of the test is another important consideration. In 2009, Lansdorp-Vogelaar and colleagues concluded that investigations such as endoscopy, FOBT, and FIT were all cost-effective given the high cost of treating late-stage CRC [32]. Ladabaum and colleagues who investigated the cost-effectiveness of SEPT9 as a screening test in Germany found that although it is more cost-effective than no screening at all, it is less cost-effective than FIT [33]. It is important to note that this model was based on a prospective study on asymptomatic patients, which showed lower sensitivity as we have discussed above. These authors have not found any studies investigating the cost-effectiveness of all the other markers identified in this study as promising in diagnosis of CRC. It is important to consider the combination of biomarkers or even combination of blood test and stool-based tests, to increase the accuracy of the test.

Among the limitations of this review is the exclusion of studies that looked at diagnostic markers for patients with adenomas or polyps but no CRC. Moreover, not all studies included all relevant demographic details on patients, whether the tumors were colonic (right versus left-sided) or rectal which can underestimate the diagnostic potential of the markers. Even with studies assessing the same marker, the assay method and cutoff values were not always homogeneous, thus introducing more variability in the results.

## Conclusions

The race is still on to discover a sensitive, specific blood-based test for the diagnosis of CRC.
